# Hockey youth in Canada: Parents’ and coaches’ beliefs towards the half-ice game model for U9 hockey

**DOI:** 10.1371/journal.pone.0305750

**Published:** 2024-06-24

**Authors:** Jean Lemoyne, Charles-Antoine Tardif

**Affiliations:** 1 Department of Human Kinetics, Université du Québec à Trois-Rivières, Trois-Rivières, QC, Canada; 2 Laboratoire de Recherche sur le Hockey UQTR, Trois-Rivières, QC, Canada; University Hospital Zurich, SWITZERLAND

## Abstract

Despite the advantages of small area games in youth sport, some challenges remain regarding the implementation of the half-ice gameplay model in Canada. In youth sport, establishing a good line of communication between parents and coaches is a crucial step for a positive environment. The purpose of this study is to provide further knowledge on the mechanisms associated with parents’ and coaches’ perceptions regarding the half-ice model in Canada. Data came from a national survey distributed across Canada (N = 6 372). Parents and coaches completed questionnaires that assessed attitudes, norms and perceived facilitators-obstacles to half-ice hockey. Parents-coaches’ preferences towards the playing format and sociodemographic variables were also measured. Structural equation modelling was performed to verify associations between each variable. Beliefs were a key factor in parents-coaches’ preferences regarding the playing format. Previous sport background and knowledge about half-ice hockey were associated with favorable predispositions. Hockey associations administrators should consider parents and coaches’ predispositions in program implementation and should design promotional campaigns adapted to their members’ predispositions towards half-ice hockey. This research underlines the key factors to consider in successful program implementation in youth sport.

## Introduction

Ice hockey is Canada’s national winter sport and reflects an important symbol of national identity [1]. With more than 500,000 registered members [2], Hockey Canada is the governing body of the nation’s largest sport federation, which make it among world’s largest ice hockey organization. With multiple success at the international level, and by developing the planet’s best hockey players, the culture of ice hockey in Canada is well established. Such context has considerable effects on the promotion of hockey among youth (e.g., children and adolescents). In this regard, Canadians, no matter where they come from, get in touch with ice hockey at a young age. The substantial socioeconomic impacts of organized hockey [3] and Canadians’ participation in different tournaments and championships [4] confirm hockey’s importance in the country [5]. Despite its status, Canadian minor hockey also has its darker side. As many authors suggest, lack of enjoyment, inappropriate parental behaviors, pressure from coaches and overspecialization in the early stages of participation are key factors that lead children to leave the game well before their teenage years [6]. This context prompted stakeholders to offer a more positive environment prioritizing enjoyment and long-term development to keep children playing. Since then, a few initiatives such as *First Shift* (https://www.firstshift.ca/the-program/) were implemented to promote positive experiences in youth hockey. In fact, stakeholders believe that creating an environment that promotes a positive experience might contribute to keep children involved in ice hockey in a positive, constructive way.

Since 2019, Hockey Canada officials adopt a shift of paradigm by revising their strategic plan and by aligning it with the Long-Term Athlete-Participant Development Model (LTAPD) [7]. The LTAPD emphasizes the acquisition of fundamental skills and promotion of enjoyment in the early stages of participation [8]. To fulfill this program orientation, Hockey Canada implemented the cross-ice development program in their Initiation (for under 7 years players) program in 2017 and decided to introduce, in 2019–2020, the half-ice gameplay model for the novice (now named U9) category [9]. Table 1 indicates the characteristics of each gameplay model, by comparing with the traditional 5 vs 5 full ice model, which is the actual format in competitive (or adult) ice hockey.

**Table 1:** A comparative overview of traditional, half-ice hockey and cross-ice hockey.

	Full-ice	Half-ice	Cross-ice (H2, H3)
**General** Playing surface (length x width)	60.96 m x 25.9 m (full rink)	30.48 m x 25.9 m	25.9 m x 22.86 m or 25.9 m x 30.48 m (using the width of the ice)
Number of players	5v5	4v4	4v4 or 3v3
Playing time	2 x 25 minutes or 3 x 15 minutes	2 periods, running time not to exceed 60 minutes total.	2 periods of 16–25 minutes, stop-time at the 2-minute mark)
Score	Displayed, recorded	Not displayed, not recorded	Not displayed, not recorded
Line changes	During play	Every two minutes	During play
**Rules**
Offside and icing	Traditionnal offside / icing rules.	No offsides / icings.	No offsides / icings.
Penalty	Offending player sit out the next shift and the team will play short-handed for the remainder of the shift.	Offending player sit out the next shift, but the team will play even strength	Penalty shots (eg., shootout) are allowed for the player that draw the penalty
Faceoffs	At the beginning of each period.	At the beginning of each period.	After each goal

Empirical support seems to go in favor of cross-ice and half-ice hockey (https://www.hockeycanada.ca/en-ca/hockey-programs/coaching/under-7). At first glance, the introduction of cross-ice and half-ice hockey appeared to be in line with more recent advances in the field of sport development. According to Peric [10], a pedagogical approach that involves adapting the playing surface to participants’ age and abilities should be prioritized, especially if the objective is about learning the game of hockey. In line with such hypotheses, an observational study conducted by USA Hockey showed positive outcomes related with cross-ice hockey; more time on ice, puck touches, shooting opportunities and passes resulted in more engagement by players [11]. Similar observations were likewise reported among cohorts of Scandinavian youth hockey players [12, 13].

### The challenges behind organization changes

Such advantages provide a good rationale that supports Hockey Canada’s decision to introduce the half-ice gameplay model for the “under 9” age-group category (U9). However, implementing organizational changes in sports institutions such as Hockey Canada brings many challenges, that bring obstacles to the process of adopting such changes. According to the Integrative Model of Organizational Change, [14], proposing a new template in an organization (e.g., sport) has the potential to affect institutional practices, by changing the norms and creating situations perceived as chaotic. In fact, even if the proposed changes seemed to be “the correct way” of doing things, “deinstitutionalize” sport organizations might lead to conflictual situations [15].

Some examples of the steps towards desinstutionalization of sport organizations exist. In this regard, the implementation of the LTADP in the scene of Canadian organized sport [16–18] underlined the challenges related with organizational changes. In this case, even if the LTADP model was theoretically favorable to long-term sport participation, its introduction in organized sport raised incomprehension and conflictual situations among youth sport stakeholders (e.g., coaches, decision makers, parents, etc.). Similar situation was observed when the Ontario Soccer Association tried to adjust their youth leagues structures [19]. Legg and colleagues concluded that it was crucial to work on establish a good line of communication with parents, coaches and decision makers to facilitate the implementation of change. From this perspective it becomes crucial to have a deeper understanding what people of importance (e.g., coaches, parents, etc.) believe about their children-players environment.

More specifically to the domain of youth hockey, changing the culture of Canada’s national sport has the potential to nurture incongruence among stakeholders’ perceptions. Considering the rich culture of ice hockey in Canada, introducing a new template for youth hockey affects the institutionalized perspectives of the sport organization. From this perspective, the introduction of the half-ice gameplay model for U9 hockey already raised debate to a certain point. In the United States, some conflictual situations were reported in the media, where almost 20 novice teams planned to quit the Michigan Amateur Hockey Association league to join full-ice leagues [20]. In Canada, the press media also brought attention to the controversy of half-ice hockey [21–23] and might raise similar kind of controversy.

In parallel to the medias’ attention, the controversy around introducing half-ice hockey also raised scholars’ interests regarding the challenges related with this organizational change. Some factors such as resistance to change, organizational constraints were cited as important barriers for the adoption of cross-ice hockey in the province of Ontario [15, 24]. A national survey led by Tardif and Lemoyne [25] revealed that parents’ and coaches’ beliefs were mitigated regarding the impacts of the U9 program. They showed that perceptions surrounding the impacts of the half-ice gameplay model differed largely across the country and, from this perspective, there could be room for “province-adapted” conflict management or implementation strategies. As suggested by Hemphill et al. [26], conflict situations are common in youth sport and can be avoided by establishing an effective line of communication between the persons involved, that is, parents and coaches. In line with Hemphill [26], Cunningham and colleagues [14] postulate that the ability of an organization (or its stakeholders) to adopt (or cope with) changes is determined by many affective normative factors. Toward this end, we think that a deeper understanding of parents’ and coaches’ beliefs and opinions regarding the half-ice game play model is crucial to evaluate the readiness of an organization to face such changes. In addition, it provides opportunity for concerted efforts (associations-parents-coaches) to implement changes in an efficient manner.

Knowing that some ambivalence related with changing a sport culture is palpable among sport organization’s stakeholders, which actions are more efficient to facilitate and organization’s adoption of change? As Riehl showed [15], the organization’s ability to change goes through multiple types of actions. For example, promoting positive outcomes (e.g., selling the benefits, promoting success stories) of the new template might be a way to convince people that the new model might be beneficial for its participants. In this regard, educating stakeholders figure as a key action. As reported by the same authors, educating stakeholders can take different forms, such as valuing beliefs about the benefits of half-ice hockey, highlighting success stories from adopters of the model and changing the norm and supporting organizations in the implementation. In other words, it becomes important to identify what factors make people apprehend change or inversely, make them favorable to them.

### Understanding the adoption of organizational change

Predispositions towards a behavior take root from complex interactions between various factors. Different theoretical models from social and educational psychology explain the importance of such factors, making it possible to develop educational interventions that might influence people positively towards a desired behavior. One of the most frequently studied models is the Theory of Planned Behavior [27]. The TPB, which inspired the present study, suggests that the intention is the predominant factor associated with the adoption of a behavior. Interestingly, the TPB was used frequently in the domain of youth sport, such as understanding parents’ intentions related with their children sport participation [28] and their intentions to support physical activity among children with disabilities [29].

The TPB proposes that an individual’s intentions are rooted in three categories of determinants: attitudinal beliefs, social norms, and perceived control over a specific action. In TPB, Attitudes refer to the degree of affect or importance accorded an object. In the present case, parents might feel unfavorable towards a new model for hockey, simply because they think that the model will not be favorable for their children’s experience in hockey (less enjoyable, not representing the sport, etc.). The second determinant of intentions is normative beliefs, which reflect the perceived social influence of behavior. Normative beliefs refer to how people feel themselves to be influenced by different groups. For example, parents could be favourably (or not) convinced by the opinion of different groups of persons (e.g., retired professional players, coaches, etc.), which might affect their intentions regarding enrolling their children in a new sport program. The third determinant of intentions, control beliefs, refer to factors perceived to be facilitators or threats regarding implementation of a behavior.

Finally, TPB stipulates that these three categories of determinants are influenced by background factors and can affect people’s perceptions and beliefs. Background factors are related with people’s beliefs about a specific object (or behavior). These factors are multifaceted and may derive from different sources; they play a crucial role in how people develop specific beliefs related to specific behaviors. Following discussions with governing bodies from the federation, and the crucial role of parents and coaches in youth participation in sport, we considered four areas of background factors that might be related with TPB constructs. identify

The first category background factor refers to parents’ background in hockey. According to Knight et al. [30], parents’ past experiences in sport characterized the way they will influence their children in sport participation. The second factor is parents’ and coaches’ previous experiences with the half-ice gameplay model. In fact, Knight [30] showed that knowledge related with ice hockey might define how stakeholders will be inclined (or not) towards adoption of the sport. Thirdly, children’s playing level is also a factor that might influence parents’ and coaches’predispositions towards a new way to the sport. In line with the Wigfield and Snelgrove [31] case study, we think that the sport culture (and consequently the resistance to change) might be stronger in more competitive environments. Finally, the stakeholders’ role (e.g., parent or coach) is a key element to consider, especially because they might perceive the children’s sport experience differently.

**Fig 1:**
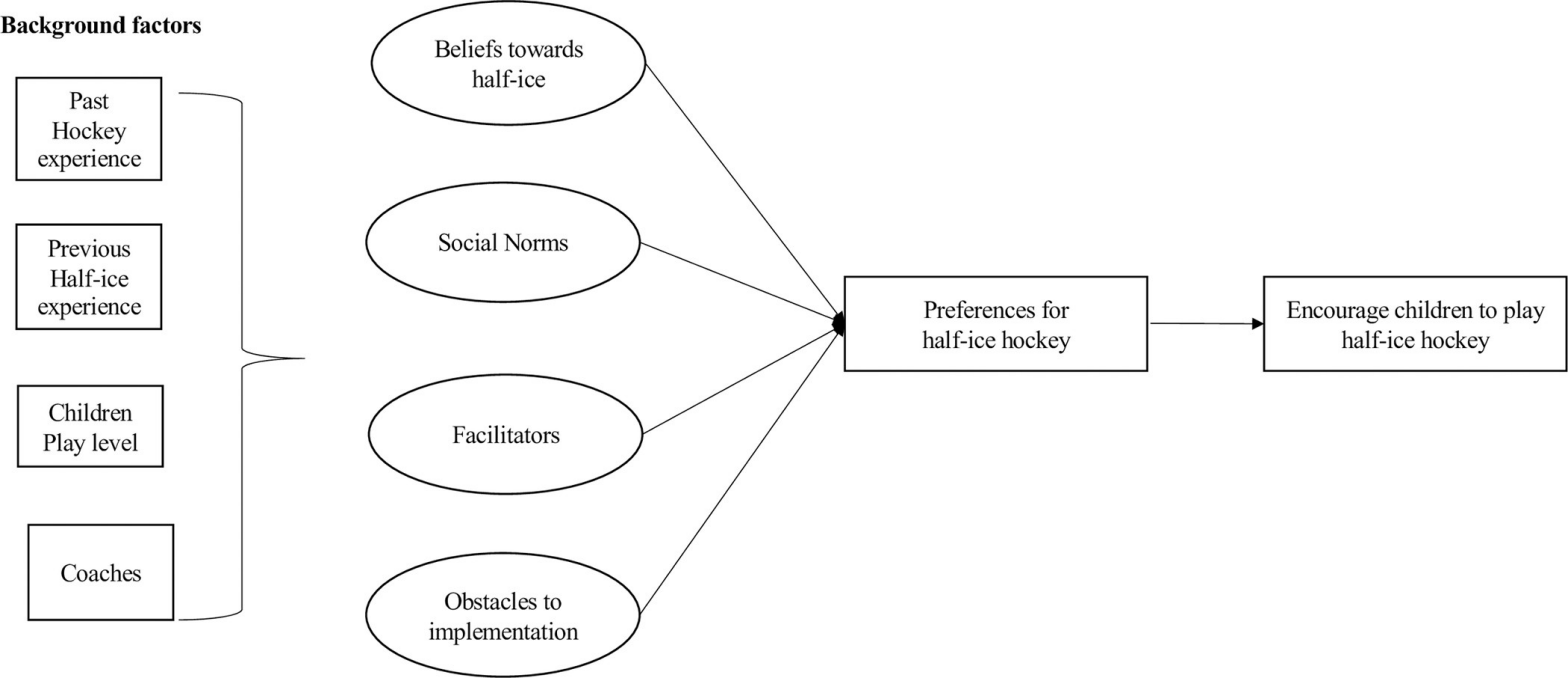
Using the TPB to explain parents’ and coaches’ predispositions towards half-ice gameplay model for U9 hockey.

Fig 1 illustrates how TPB is operationalized in the context of the implementation of a new gameplay model for youth hockey (e.g., half-ice). From there, we conceptualized the intention construct as parents’ and coaches’ preferences towards two playing formats: the 5 vs 5 model (e.g., traditional, full ice hockey) and the half-ice gameplay model. An example in the present case is to see how parents and coaches believe half-ice hockey to be of benefit to their children or the players. As mentioned earlier, past research showed that parents and coaches tend to be ambivalent regarding different categories of beliefs associated with half-ice hockey [25]. In line with organizational change theories, educating stakeholders towards favorable beliefs could be the start of a smooth transition from traditional hockey to the half-ice play model.

### Objectives of the study

The aim of this study is to provide a deeper understanding of the mechanisms associated with parents’ and coaches’ perceptions of the half-ice game play model in Canada and their preferences for 5 vs 5, full-ice hockey. More specifically, we aim to identify the variables associated with their preferences towards the half-ice play model as opposed to the traditional form of hockey (5 vs 5). Second, this study analyzes the role of four background factors that may be associated with parents’ and coaches’ perceptions of the half-ice game play model: 1) their past experiences as a hockey player, 2) having children-players who had previous experience with the half-ice initiative, 3) children-players’ playing level and 4) their role as parent or coach. We feel that a better understanding of the mechanisms associated with the half-ice model will facilitate its implementation across Canada in different ways: first, by sensitizing parents (to key determinants) and second, by reinforcing some key aspects of coach education for coaches involved in the early stages of participation.

This study was designed to serve as a baseline measure for facilitating half-ice implementation in Canada and guide future work [25]. In addition, these measures should serve as baseline measures to verify the evolution of beliefs across time, following the implementation of the half-ice gameplay model. At a practical level, an improved knowledge of these mechanisms could be useful for stakeholders seeking to develop effective approaches to sensitize, educate and assist parents during implementation of the half-ice program by offering the appropriate resources.

## Method

### Participants and procedures

Data for this study came from a survey conducted among 6 372 participants; 81% were parents of novice hockey players (n = 5 130) and 19% were coaches working with novice hockey teams (n = 1 242). We obtained the collaboration of Hockey Canada for recruitment of participants, and a convenience sample was used based on email invitations to members (through provincial branches). Participants came from nine provinces and territories (British Columbia, Alberta, Saskatchewan, Ontario, Quebec, Newfoundland, New Brunswick, Prince Edward Island and Nova Scotia). Participants were invited to participate through email invitations. Informed consent form (sent by email) was completed on-line by parents and coaches (e.g., by checking the case that mentioned “*I agree to take part to the survey*”). This research was approved by the human research ethics board of the institution involved in the research (CER-17-239-07.08). The data were collected in the 2018–2019 season (November 2018—May 2019), one year prior to implementation of the half-ice play model for novice players across Canada.

### Measures

The survey was developed in an elicitation phase involving the Hockey Canada Board of directors and regional branch members. The questionnaire was designed based on the TPB framework and in keeping with the recommendations of Francis et al. [31]. A first version of the questionnaire was revised by Hockey Canada and Hockey Quebec board members for purposes of clarity; it was then pre-tested with the expert committee. Then, we validated the content in both languages (French and English) and reformatted some items to ensure the meaning was the same in both languages. Parents’ and coaches’ questionnaires were identical, except for the wording of certain items (the term “*my children*” was replaced by “*my players*”). The questionnaire (S1) included a total of 35 items evaluating six constructs: 1) beliefs related to half-ice hockey (13 items), 2) normative beliefs (8 items), 3) facilitating factors (4 items), 4) obstacles to half-ice hockey (4 items), 5) background factors (4 items) and 6) parents’ and coaches’ preferences regarding novice hockey (2 items). As to preferences regarding both types of game play models, we asked parents and coaches in which model they wanted to see their children evolve (half-ice and 5-on-5 full-ice). All sub-scales were scored according to a 5-point Likert scale (1 = totally disagree, = somewhat disagree, 3 = uncertain, 4 = somewhat agree, 5 = totally agree).

#### Attitudes-beliefs regarding half-ice hockey

We used a 13-item sub-scale to measure parents’ and coaches’ beliefs. For each item, participants were asked: “*In your opinion*, *how is half-ice better than 5 vs 5 hockey*” (see Table 2). The principal component analysis (with varimax rotation, eigen values fixed at 1.0) was conducted with the full sample and revealed a two-dimension structure. Next, we specified the belief factor using two indicators: the “hockey development” sub-scale consisted of 10 items related to beliefs regarding the benefits of half-ice hockey (σ = 0.96; ICC = 0.72), and the “hockey specific” sub-scale included 3 items and showed good reliability (σ = 0.86; ICC = 0.65).

**Table 2:** Participants’ profile (proportions of participants).

	Total (n = 6 372)	Parents (n = 5 130)	Coaches (n = 1 242)
Gender (M-F)	n/a	50% - 50%	n/a
Past sport experiences	> 85%	91% - 75%	96%
Have experience in half-ice play model	83%	75%	95%
Children-players in competitive league	28%	n/a	n/a

M; Mothers; F: Fathers

#### Social norms

We used an 8-item sub-scale to measure social norms (see Table 2). For each item, participants were asked: “*How do you think these groups of persons might influence you towards half-ice hockey*?” The principal component analysis suggested that social norms were identified as a unidimensional factor. Reliability analyses revealed very good reliability for the social norms scale (σ = 0.90; ICC = 0.53).

#### Facilitators and barriers

We used an 8-item sub-scale to evaluate facilitators (4) and perceived barriers (4) to half-ice hockey (See Table 2). For each item, participants were asked: “*How do you think these factors would (facilitate-complicate) the implementation of half-ice hockey in your association*?” As expected, the principal component analysis suggested a two-dimensional structure: facilitators and barriers. A high facilitators score would mean that such factor is viewed as an important, favorable influence; inversely, a high barriers score would mean that it is viewed as a barrier to implementation. Reliability analyses revealed very good reliability for both sub-scales (Facilitators: σ = 0.83; ICC = 0.54; Barriers: σ = 0.72; ICC = 0.39).

#### Parents’ and coaches’ preferences regarding the play model

Two single items were used to measure parents’ and coaches’ preferences regarding the play format of children and players. We used the same 5-pt Likert scale and asked participants two questions: 1) If I have a choice, I would prefer see my children-players evolve in traditional 5-on-5 hockey, and 2) I prefer to see my children-players evolve in traditional half-ice hockey.

#### Background factors

We measured four background factors by questioning parents and coaches on the basis of four factors: 1) previous hockey experience, 2) previous experience with the half-ice initiative (as parent or coach), 3) children’s and players’ playing level, and 4) participants’ role (e.g., parent or coach). We evaluated participants’ past experience in organized hockey by asking if “*they played organized hockey in the past*” (no = 0; yes = 1). As for their experience with the half-ice game model, we asked “*if there was a half-ice initiative in your hockey associations*” (no = 0; yes = 1). We measured children’s playing level by asking parents and coaches to select “children’s or players’ level of play” (competitive-rep leagues = 1; recreational-community leagues = 2). Participants’ role was defined based on the role they play in their children’s hockey participation. Three options were possible: parent-only; parent-coach; coach-only. Because there were no significant differences between parents-only and parent-coaches (Tardif & Lemoyne, 2021), the participants’ role was divided into two categories (parents = 1; coaches-only = 2).

### Statistical analyses

#### Missing data handling and multivariate normality

All analyses were performed with Mplus (version 8.5) software [32]. We handled missing data by using the expectation maximization algorithm (EM: the default option in Mplus). This approach is common with large samples having less than 10% of missing data [33]. We tested multivariate normality by computing the Mardia coefficient, which revealed violation of multivariate normality assumptions (Skewness test = 6.969; Kurtosis test =. 131.080, both at *p* < .001). Based on these results, we conducted a model estimation using the maximum likelihood with robust estimates.

#### Model testing

We used structural equation modeling (SEM) to perform path analyses. This procedure has many advantages, including the measurement of latent variables and the availability of measures of global fit that can provide a summary evaluation of complex models involving a large number of parameters. We tested three models, including all constructs that could be associated with parents’ and coaches’ intentions regarding both game play models (half-ice and traditional 5-on-5). In Model 1, we assumed that beliefs, social influences and perceived control would be the three determinants of parents’ and coaches’ preferences (modelized as intentions). In Model 2, we added background factors to Model 1 and modelized them as potential influences on parents’ and coaches’ beliefs, social influences and perceived control. In Model 3, we tested the indirect effects of background factors on parents’ and coaches’ preferences regarding both game play models. Significant effects would indicate that background factors play a role in the relationships between the three main determinants and preferences of a game play model. We evaluated each model by using the most common fit indices: chi-square coefficient (χ2), comparative fit index (CFI), Tucker-Lewis index (TLI) and the root mean square error of approximation (RMSEA). Interpretation was based on cut-off values suggested by Hu and Bentler [34]: 1) chi-square (χ2) value as a global estimation of fit, 2) CFI and TLI (values > .95), 3) Root Mean Square Error Approximation (RMSEA), and 4) Standardized Root Mean Residual (SRMR) (< .07). Given the chi-square statistic is highly sensitive to sample size (n > 1 000), we followed Kline’s recommendations by inspecting the residual correlation matrix [35]. Values over .10 would be interpreted as problematic and need to be analyzed to see if their correspondence is a threat for model fit.

## Results

### Sample characteristics

Table 2 illustrates the participants’ profile. In the overall sample, 81% of participants are parents and the remaining 19% are coaches only. They came from eight different provinces-territories. A total of 67% of the participants (n = 4 265) had previous experience in ice hockey as a player, and 83% report their child (or players) had previous experience with the half-ice play model (n = 5 272). Almost 30% report having a child (or players) who played in more competitive leagues (called “rep” leagues) compared with 72% who played in community leagues (recreational).

### Model 1. Determining parents’ and coaches’ preferences regarding play models

Analyses from Model 1 revealed very good fit indices (CFI = .987; TLI = .980; RMSEA = .037, *p* = 1.00) with the exception of the chi-square statistic (χ^2^_(df)_ = 533.37_(57)_; *p* < .001). Although the chi-square can be affected by sample size, we observed residual correlations and found only three associations exceeding the 0.1 cut-off value (involving some facilitator and barrier items). As Table 3 shows, all constructs were significantly correlated.

**Table 3:** Correlation matrix (results from model 1).

Construct	Beliefs	Social influences	Facilitators	Barriers
Beliefs	1.0			
Social influences	.769	1.0		
Facilitators to half-ice	.776	.764	1.0	
Barriers to half-ice	-.462	-.308	-.292	1.0

All correlations significant at *p* < .001

Table 4 shows factor loadings for Model 1. Favorable beliefs regarding the impacts of half-ice hockey were positively associated with parents’ and coaches’ preferences towards the half-ice game play model (ϕ = 1.061, *p* < .001), whereas they were negatively (ϕ = -0.989, *p* < .001) associated with their preferences for 5-vs-5 hockey. Social norms were positively associated with preferences towards 5-vs-5 hockey (ϕ = 0.082, *p* < .001), indicating that different types of social agents are crucial to convince those more in favour of traditional hockey. However, perceived facilitators and barriers to half-ice implementation were less important (e.g., non-significant, negative scores) among those demonstrating a preference for half-ice hockey compared with those who prefer traditional 5-vs-5 hockey. Model 1 explains 84% of parents’ and coaches’ preferences for half-ice game play, and 73% of preferences for the traditional 5-vs-5 full-ice model.

**Table 4:** Model 1: Parents’ and coaches’ preferences towards novice hockey game play models (standardized parameter estimates).

Latent factor	Indicator	Mean ±Variance (loading)	→ Half [SE]	→ Full (SE)	R^2^
Beliefs towards half-ice	B1	2.88 ± 1.25 (.96)	1.061** [0.015]	-0.989** [0.016]	.92** .60**
	B2	2.23 ± 1.01 (.78)			
Normative beliefs	N1	3.25 ± 1.11 (.88)	-0.048 [0.015]	0.082** [0.017]	.78** .66**
	N2	2.80 ± 1.18 (.81)			
Facilitators	Fa1	3.57 ± 1.36 (.76)	-0.165* [0.016]	0.159** [0.017]	.58**
	Fa2	3.37 ± 1.57 (.79)			.62**
	Fa3	3.59 ± 1.53 (.87)			.76**
	Fa4	3.62 ± 1.71 (.53)			.28**
Barriers	Ba1	2.95 ± 1.42 (.53)	-0.022* [0.008]	0.075** [0.011]	.26**
	Ba2	2.89 ± 1.86 (.98)			.60**
	Ba3	2,88 ± 1.59 (.10)			.02 *
	Ba4	2.55 ± 1.35 (.69)			.40**
Preferences (half-ice)	n/a	2.64 ± 2.45	N/A	N/A	.84**
Preferences (full-ice)	n/a	3.55 ± 2.09	N/A	N/A	.73**

*p < .05

**p < .001; [SE]: standard error of estimates

### Model 2. Role of background factors on parents-coaches’ beliefs

Analyses from Model 2 also reveal good fit indices (CFI = .982; TLI = .972; RMSEA = .036, *p* = 1.00) with the exception of the chi-square statistic (χ^2^_(df)_ = 878.34_(95)_; *p* < .001). As Table 5 indicates, parents and coaches with previous experience in organized hockey had more favorable beliefs towards half-ice hockey (ϕ_beliefs_ = 0.027, *p* < .05) and less influence from social norms (ϕ_norms_ = -0.053, *p* < .001) and facilitators (ϕ_facil_ = -0.043, *p* < .001). In addition, previous experience in organized hockey was positively associated with preferences for the half-ice game play model (γ = 0.030, p < .001). Parents and coaches with children-players having previous half-ice game play experience constituted a key determinant of beliefs in favour of adopting half-ice hockey (ϕ = 0.210, *p* < .001), social norms (ϕ = 0.149, *p* < .001), facilitators (ϕ = 0.138, *p* < .001), and barriers (ϕ = -0.176, *p* < .001). Past experience was also positively associated with preferences regarding the half-ice gameplay model (γ = 0.026, *p* < .001). Parents and coaches of children-players who evolved in more competitive leagues (e.g., “rep” league) tend to be less favorable to half-ice hockey (all coefficients were negative and significant at *p* < .001) and perceive greater barriers to implementation (ϕ = 0.034, *p* < .05). Indeed, the playing level of children-players factor was not significantly associated with preferences for half-ice hockey. With respect to one’s role, coaches tend to report more favorable beliefs (ϕ = 0.054, *p* < .001), were inclined to act according to social norms (ϕ = 0.029, *p* < .001) and were more sensitive to facilitating factors (ϕ = 0.048, *p* < .001).

**Table 5:** Model 2 background factors as determinants of beliefs.

Background factors	Beliefs	Norms	Facilitators	Barriers	Association with half-ice
Previous hockey experience	0.027* [.013]	-0.053** [.014]	-0.043** [.014]	-0.030^ns^ [.015]	0.030* favorable
Having a half-ice experience	0.210** [.012]	0.149** [.014]	0.138** [.014]	-0.176** [.014]	0.026** favorable
Playing at competitive level	-0.091** [.012]	-0.072** [.014]	-0.043** [.014]	0.034* [.014]	ns *unfavorable*
Coach	0.054** [.012]	0.029* [.014]	0.048** [.013]	0.000^ns^ [.014]	ns *coaches > parents*

*p < .05

**p < .01

### Model 3. Introducing background factors to explain parents’ and coaches’ preferences for half-ice hockey: the indirect effects

**Fig 2:**
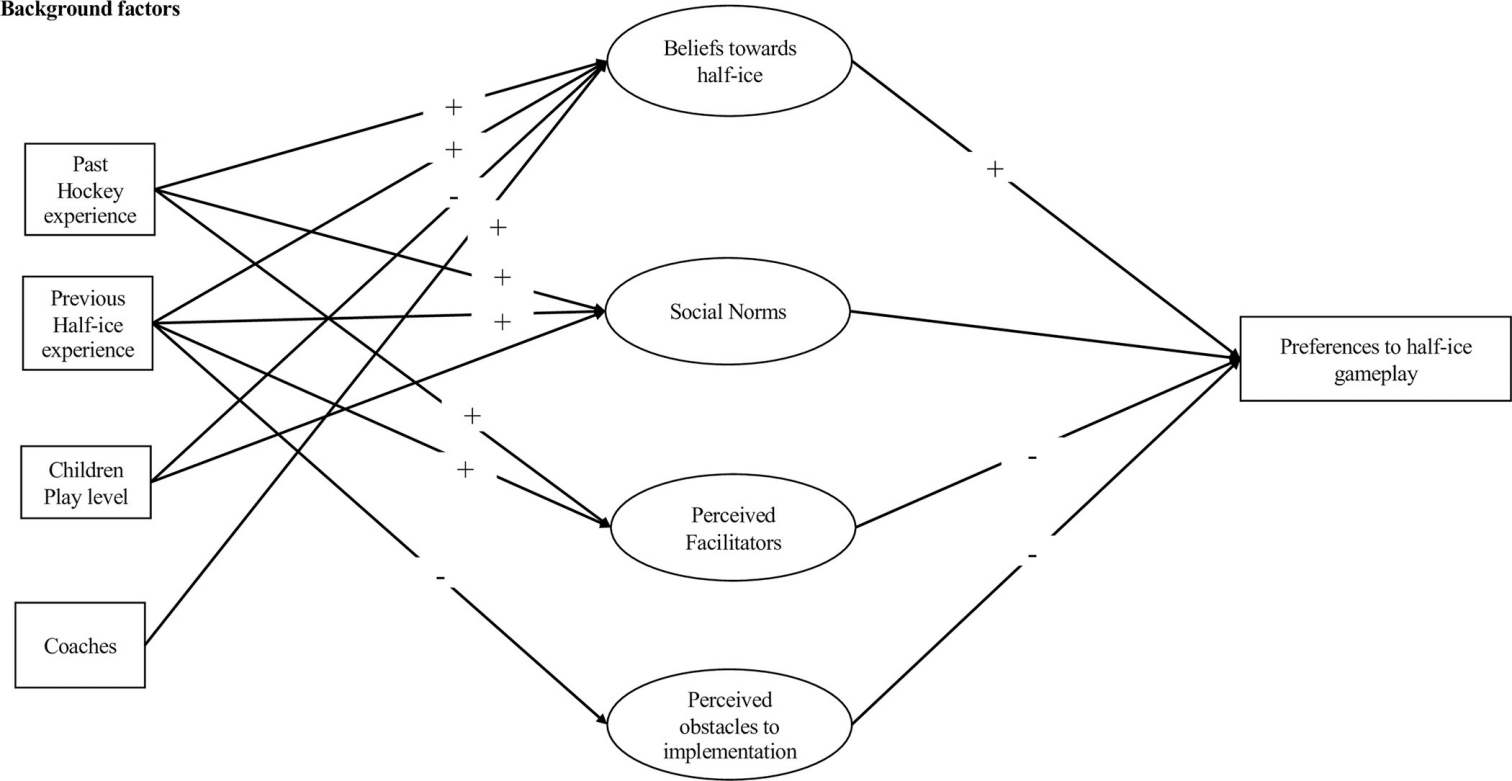
Understanding parents’ and coaches’ preferences towards the half-ice model for novice hockey.

Fig 2 illustrated Model 3, which explains parents’ and coaches’ preferences regarding the half-ice game play model for novice hockey. We tested the indirect effects to confirm if background factors associated with parents’ and coaches’ preferences were mediated by beliefs, social norms, facilitators and perceived barriers related to half-ice hockey. As Fig 2 shows, the effect of parents and coaches’ previous experiences in organized hockey (e.g., as a player) was mediated through beliefs, norms and facilitators, suggesting that past experience in organized hockey is associated with positive feelings towards half-ice hockey. Previous experience with the half-ice game play initiative was mediated by all variables, which suggests this factor is a positive contribution. Children’s playing level was mediated by beliefs, which means that parents and coaches whose children-players evolve at more competitive levels were less inclined towards the half-ice game play model; however, despite unfavorable beliefs in this regard, they seemed more open to influenced favourably by social agents such as other parents, coaches and authority figures in their federation or local association. As for the participants’ role, that of coach was mediated by favorable beliefs towards half-ice hockey, and lesser importance was given to social influences and facilitators regarding implementation.

## Discussion

Establishing a healthy and positive climate for youth sport participation is a key element in promoting long-term sport participation and requires increased emphasis on play patterns that allow for enjoyment and youth development. Hockey Canada’s initiative of implementing the half-ice gameplay model for U9 hockey is a substantial organizational change, considering the rich culture of hockey in Canada. In line with recommendations from past research, knowing more about the mechanisms that lead to favorable intentions towards adoption of the half-ice gameplay model is a key step for facilitating organizational change. As mentioned by Riehl, Snelgrove and Edwards [15], modifying tradition in an established sport like hockey in Canada require a deep understanding of the factors that might contribute to connect decision makers with stakeholders (e.g., hockey association directors, coaches, parents) who are directly involved in the paradigm shift. Moreover, by taking account of parents’ and coaches’ beliefs, because they are the key actors in establishing to sport initiation environment. In this regard, this study suggests interesting theoretical and practical insights regarding the factors to consider to consider when trying to implement a positive sport environment.

This study is a first step towards laying the groundwork for an educational perspective that will facilitate implementation through a better understanding of the attitudes of Hockey Canada officials’ regarding the half-ice game play model. Results from previous work show that parents’ and coaches’ attitudes-beliefs towards the half-ice gameplay model vary importantly across Canada [25]. As postulated by Tardif and Lemoyne, some of these differences can be explained in part by people’s familiarity with the half-ice play game model, since many provincial associations already had introduced the half-ice model in their associations, at the time of data collection. In the present study, therefore, we decided to go further by exploring the contribution of additional variables in an effort to shed a light on mechanisms that could facilitate implementation across the country. In line with the integrative model for changes in sport organizations [14], understanding stakeholders’ beliefs, norms and opinions towards change is a key step in the process of “reinstitutionalization” of the sport. In this regard, using the TPB framework as the theoretical foundation of this study was promising, more specifically because it allows for the inclusion of beliefs, social influences, perceived barriers and other factors likely to influence parents’ and coaches’ preferences towards change. TPB was used in previous sport studies, and we think that its practical perspective can guide for designing interventions in the future.

The results of this study demonstrate that parents’ and coaches’ preferences for the half-ice gameplay model are based on beliefs about perceived benefits for the child. In fact, parents and coaches will tend to display favorable perceptions with the program if they are convinced that this model is more fun and is more beneficial for learning hockey, which consequently to the intentions to register their child in such program. As Howe and Krosnick stipulate, attitude strength is a crucial factor associated with people’s beliefs towards an object and, consequently, their willingness to adopt the behavior [36]. In this regard, educational campaigns, consciousness-raising sessions and updated coach education are key components for convincing people that half-ice hockey is better for novice player development. A campaign such as this might promote global benefits of the half-ice gameplay model, by putting emphasis on the enjoyment and positive experiences and combining with the positive learning experiences that comes from such practice, instead of focusing specifically on the technical aspects of ice hockey (e.g., skating). However, even if the “technical” benefits of half-ice are supported by research, there appears to be less support for its positive motivational outcomes (e.g., is it really more fun?). Additional analyses conducted in this sample reveal that parents and coaches expressed some doubts about children’s enjoyment of half-ice hockey [25]. In this regard, more specific attention to this issue (e.g., affective component of change) will require further attention in a near future.

Interestingly, our results also provide support for considering the impact of social influences towards the implementation of change (e.g., introducing half-ice hockey) in hockey associations. Analyses from Model 1 suggest that, parents and coaches who privileged 5-vs-5 hockey were more susceptible to be influenced by other people towards the half-ice gameplay model. These results are somewhat surprising and interesting, suggesting that parents and coaches who already favor the half-ice game play model do not attach much importance to other persons’ convictions about choosing half-ice hockey for children-players. In fact, their choice is made, and they do not need additional sensibilization regarding the half-ice gameplay model. In contrast, parents and coaches more in favor of 5-vs-5 full-ice hockey seemed to more incline to receive advice-information from different groups of persons (e.g., their children’s coaches, other parents, different stakeholders from federations and local associations). This is encouraging and opens the door for educational and consciousness-raising initiatives that emphasize the positive outcomes of adapting the play surface for youth hockey players. Further analyses using our sample also show that coaches were perceived as the people with the higher level of influence towards convincing about the importance of encouraging children to play in half-ice hockey. From there, such results shows that coach education is an important part of the implementation process. In line with Riehl’s suggestions [15], coach education could be one of those actions that lead to more favorable perceptions towards change. Similar tendencies were observed when we analyzed facilitators and perceived barriers on parents’ and coaches’ game play preferences. First, those in favor of the traditional 5-on-5 gameplay noted many facilitating factors and perceived a greater number of barriers. Inversely, parents and coaches that were incline to the half-ice gameplay model perceived less barriers and accorded less importance to facilitating factors. In fact, it may be that those in the “favorable” sample had some experience with the half-ice game play model, which explains the lower regression coefficients. On the other hand, parents and coaches with less experience and knowledge regarding introduction of the program to local hockey associations may view certain challenges as major barriers to implementation. Furthermore, our data show that the half-ice experience is no guarantee that parents and coaches will adopt half-ice hockey. We mentioned earlier that almost 40% of those in this sample reported they prefer the traditional (5-vs-5) game play format. This indicates that hockey culture is still strong in Canada and the introduction of new ways of playing the game will encounter many challenges.

As already explained, it appears that previous experience and knowledge regarding the half-ice game play model positively influences attitudes towards the program. This result is encouraging, since parents and coaches, although ambivalent in some respects, seem favorably inclined once the experience is underway. Similar conclusions were observed in youth hockey, when body check rules [37] implemented for U15 players and officials introduced mouthguards in categories allowing physical contact [38]. It is also encouraging to observe that coaches and parents with previous experience as hockey players were favourably inclined towards the half-ice game play model. A possible side effect of past experience is the crystallization of attitudes and beliefs in certain situations. However, our results show that those who had already played organized hockey did not view half-ice implementation as detrimental to the culture of Canada’s national sport. Thus, they could positively influence the implementation of the program across Canada by sharing their past experiences and relating them to the benefits of introducing half-ice hockey during the early years of participation.

In line with these results, it is likewise encouraging to see that coaches tend to view the half-ice game play model positively, which is promising for a smooth transition from full-ice 5-on-5 to half-ice play. However, our results confirm that children’s playing level is a key variable that demands greater attention. In fact, our analyses reveal that the parents and coaches of children playing at the most competitive levels (e.g., known as “rep” league, Novice A, etc.) are less convinced of the benefits of half-ice hockey, which aligns with the hypothesis suggesting that “half-ice will not be good for the best U9 players” [21–24]. Such results merit attention and need to be considered by Hockey Canada officials when introducing the program among the “more competitive” leagues. Consciousness-raising campaigns and coach education workshops emphasizing the benefits of small area games for “elite or competitive” players are needed and could help convince more competitive associations to embrace this change of culture.

### Limitations and future research

This study provides an update on the factors to be considered when implementing the half-ice hockey program in Canada. The development of a questionnaire to identify parents’ and coaches’ preferences-beliefs can potentially lead to future measures to monitor changes in beliefs over time (e.g., first years of implementation). Despite its contribution and promising results, this study also has its share of limitations. First, the sample, while large, does not systematically eliminate a possible selection bias. As evidenced by our data, over 75% of respondents reported knowing or having seen children and players who experienced the half-ice game play model. Although the data were collected at the start of implementation, it’s possible that many participants who did not experience the initiative chose not to participate in the study.

A second limitation concerns the questionnaire itself, which we believe may not capture all the beliefs related to facilitating factors and perceived barriers. These two sub-scales were constructed prior to the implementation of the half-ice program, based on situations that were anticipated before implementation [25]. We therefore believe that a follow-up with stakeholders using semi-structured interviews could be useful for updating or adjusting this part of the questionnaire. A third limitation relates to the questions parents and coaches were asked regarding their beliefs and attitudes towards the half-ice game play model. Although measurement of children’s beliefs and perceptions involves significant methodological challenges, methodology must be used that provides a more representative picture of the actual experience. This measure was beyond the scope of this study however. It was necessary to start with the data from parents and coaches, particularly since they are the key to establishing a positive environment in the early stages of sport participation. In the near future, however, we plan to develop approaches allowing us to measure children’s perceptions of the half-ice experience.

## Conclusion

This study provided additional knowledge in relation with the process of organizational change for sport and leisure organizations. As the results from this investigation showed, implementing change can be facilitated by considering people’s beliefs and perceptions about an organization’s intention. This study also demonstrated that people’s perceptions might differ according to characteristics such as past experience with sport and the motives that lead them to be engage in a sport or leisure (e.g., ti competition or playing in a recreational perspective). Knowing more about such characteristics (e.g., past experience, level of expertise) might help decision makers to structure how they will implement change in their sport associations. More specific to the field of youth hockey, this study also brought some additional knowledge. First, it identifies promising avenues for future research that will help broaden our knowledge regarding the youth hockey model in Canada. As youth in Canada are now introduced to hockey in new ways, the relevance of making the half-ice game play model a positive experience is understandable. Toward this end, decision-makers need to identify resources that will facilitate its implementation in the early stages of sport participation. Considering the symbolic role of ice hockey in Canada, it is imperative to adapt the message to different communities and help promote a sport that’s accessible to all. The half-ice hockey initiative takes these aspects into consideration by offering children an environment that maximizes learning situations and offers a more gradual introduction to the sport. Specific challenges apply to associations that welcome experienced youths who seek to play in more competitive environments and leagues. It is therefore essential to adapt the message to achieve more effective implementation and prevent situations that can lead to controversy. For the researchers, the implementation of the half-ice program offers an opportunity to examine how organized sport helps develop healthy physical literacy and create a healthy environment through sport participation.

## Supporting information

S1 File (DOCX)

S2 File (DAT)
