# Supplementary material for: Hockey youth in Canada: Parents’ and coaches’ beliefs towards the half-ice game model for U9 hockey
Source: PLoS One. 2024 Jun 24;19(6):e0305750. doi: 10.1371/journal.pone.0305750 (PMC11195953; doi:10.1371/journal.pone.0305750)
Supplement: S1 File — (DOCX) [file pone.0305750.s001.docx]

| **SECTION I** **Information and consent** | |
| --- | --- |
| The**purpose of this project**is to**verify your beliefs**about the implementation of the half-ice Novice Hockey program, which will be implemented in 2019.  **This project requires a simple involvement from you:**Complete a survey about your knowledge, beliefs, practices, and perceptions towards half-ice hockey. It will take about 10 minutes to fulfill the survey.  **What is the new learning program introduced by Hockey Canada Novice?** The purpose of Hockey Canada's initiative towards novice ice hockey is to implant a new learning program adapted to participants' age. The project aims to limit the concept of competition while offering a suitable environment for motor skills development. The friendly oriented games will be played in half-ice. The program's primary objective is to offer young players the opportunity to live successes, so they can associate the feeling of pleasure to hockey (determining element for players' development).  **How is half-ice hockey organized?** Since the playing surface is smaller, the rules must be modified. For the novice players, the game is played 4 versus 4 and lasts 50 minutes (2 periods of 25 minutes) in continuous time. The changes are called by a buzzer every 60 seconds without interrupting the gameplay. There are only 2 face-offs: at the beginning of each period.  **Why do we want to make the players progress in a half-ice context?** Many organizations have already adopted restricted space in the field of different sports like soccer, tennis, basketball and baseball. It allows to adapt the gameplay and the game area to players' age.  **Voluntary contribution:** Your contribution is voluntary. Refusing to fulfill the survey does not lead to consequences.  **Risks / Benefits** There are no risks or disadvantage related to the project except the time required to fulfill the survey. By accepting, you contribute to knowledge advancements in organized ice hockey for youths.  **Confidentiality of data** All data will be processed confidentially, respecting anonymity of the participants. The results will be disclosed globally including all participants.  **This research is approved by the human research ethic comity of Université du Québec à Trois-Rivières and a certificate numbered CER-17-239-07.08 has been issued on October 25th 2017.**  For any questions or ethical complaint concerning this research, you must communicate with the research ethic comity secretary of Université du Québec à Trois-Rivières, at Deanship of graduate studies and research, by phone (819) 376-5011 #2129 or by email CEREH@uqtr.ca.  **Person in charge of the research** If you need any further details or for any question regarding this research project, you can reach:  Jean Lemoyne, PhD Professor Department of Physical Activity Sciences Université du Québec à Trois-Rivières 819-376-5011 au poste #3794 jean.lemoyne@uqtr.ca | |
| 1. By checking ''YES'', you confirm that you have read the instructions related to ethic and deontology of the research and accept to take part in the study by completing the survey. | - Yes - No |
| 2. Would you be interested to participate in a subsequent phase of the project by taking part in discussion groups related to the subject? | - Yes - No |
| 3. If you answered YES, specify by which means it would be possible to reach you with your contact information.   \| Name \|  \| \| --- \| --- \| \| Email address \|  \| \| Phone number \|  \| | |

| **SECTION II** **Demographic data and profile**  **In this section, we are interested in your branch of activity, as well as your child’s experience in competition level hockey.** | | |
| --- | --- | --- |
| 4.At which level does you child play? | - Novice A - Novice B - Novice C - Other: _____________________ | |
| 5. Did you know about the half-ice hockey learning program initiative by Hockey Canada prior to this survey? | - Yes - No | |
| 6. Is there an half-ice gameplay initiative in your area? | - Yes - No | |
| 7. You are… | - The mother of your child - The father of your child - Other | |
| 8. Province | - Alberta - British Colombia - Manitoba - New Brunswick - Newfoundland / Labrador | - Nova Scotia - Ontario - Prince Edward Island - Quebec - Saskatchewan |
| 9. Region  If outside of Quebec, please check here | - Bas St-Laurent - Côte-Nord - Estrie - Gaspésie Les Îles - Lac St-Louis - Laurentides-Lanaudière - Laval | - Mauricie - Outaouais - Chaudière-Appalaches - Montréal - Richelieu - Saguenay Lac St-Jean |
| 10. Place of residence (town):  ______________________ | 11. Postal code: ______________________ | |
| 12. In the past, have you practiced a competitive sport? | - Yes - No   If yes, which one? ___________________________ | |

| **SECTION III** **Your experience in ice hockey**  **In this section, we are interested in your experiences and perceptions related to ice hockey.** | |
| --- | --- |
| 13. Have you ever played ice hockey? | - Yes - No |
| 14. Are you involved as a hockey coach? | - Yes - No |
| 15. Are you involved as a volunteer in hockey? | - Yes - No |
| 16. Do you have another child who plays hockey? | - Yes - No |
| 17. Among the following factors and based on your opinion, indicate in order (1 to 5) the importance you attach to the situations presented below: THERE ARE NO WRONG ANSWERS. | __ Have fun practicing a sport  __ Develop technical and tactical skills  __ Initiate youths to competition  __ Prompt youths to be physically active  __ Teach youths to play a specific role/position |

| **SECTION IV Attitudes and beliefs**  **In this section, we ask you to indicate your beliefs and perceptions**  **by comparing half-ice hockey and traditional 5 on 5 hockey.** | | | | | |
| --- | --- | --- | --- | --- | --- |
| 18. Regarding my child's level of play: I think that comparatively to traditional gameplay, half-ice hockey would be... | | | | | |
|  | Total disagreement | Pretty much in disagreement | I don't know... | Pretty much in agreement | Total agreement |
| More useful to players' development | 1 | 2 | 3 | 4 | 5 |
| More motivating for players | 1 | 2 | 3 | 4 | 5 |
| More fun than 5 on 5 | 1 | 2 | 3 | 4 | 5 |
| More beneficial to learn hockey | 1 | 2 | 3 | 4 | 5 |
|  | | | | | |

| 19. Regarding my child's level of play... I think that comparatively to traditional gameplay, half-ice hockey contributes in a better way to... | | | | | |
| --- | --- | --- | --- | --- | --- |
|  | Total disagreement | Pretty much in disagreement | I don't know... | Pretty much in agreement | Total agreement |
| Develop skating skills | 1 | 2 | 3 | 4 | 5 |
| Develop technical abilities (shots, passes) | 1 | 2 | 3 | 4 | 5 |
| Develop individual tactics (1 on 1, puck protection) | 1 | 2 | 3 | 4 | 5 |
| Develop the notions of team spirit | 1 | 2 | 3 | 4 | 5 |
| Learn the game's rules | 1 | 2 | 3 | 4 | 5 |
| Learn to play a specific position | 1 | 2 | 3 | 4 | 5 |
| Allow children to be more active | 1 | 2 | 3 | 4 | 5 |
|  | | | | | |

| **SECTION V Influences and barriers**  **In this section, we evaluate the influence that people around you could have on your beliefs related to half-ice hockey. Also, we are trying to identify some barriers to establishing such a practice.** | | | | | |
| --- | --- | --- | --- | --- | --- |
| 20. At what level do you think the following are **factors that would make it easier** to set up a half-ice game program? | | | | | |
|  | Total disagreement | Pretty much in disagreement | I don't know... | Pretty much in agreement | Total agreement |
| Coaching training workshops | 1 | 2 | 3 | 4 | 5 |
| Organize a half-ice tournament | 1 | 2 | 3 | 4 | 5 |
| Raising awareness with parents | 1 | 2 | 3 | 4 | 5 |
| The decrease in registration fees related to the use of arenas (maximize use) | 1 | 2 | 3 | 4 | 5 |
|  | | | | | |

| 21. At what level do you think the following are **barriers**  that would prevent you from setting up a half-ice game program? | | | | | |
| --- | --- | --- | --- | --- | --- |
|  | Total disagreement | Pretty much in disagreement | I don't know... | Pretty much in agreement | Total agreement |
| There could be a lack of material | 1 | 2 | 3 | 4 | 5 |
| There could be too many players at once on the ice | 1 | 2 | 3 | 4 | 5 |
| Coaches may not have the required skills/training | 1 | 2 | 3 | 4 | 5 |
| Teams' players selection would be too complicated | 1 | 2 | 3 | 4 | 5 |
|  | | | | | |

| 22. If we had to convince myself... Who are the people most likely to positively influence you regarding half-ice hockey development programs? | | | | | |
| --- | --- | --- | --- | --- | --- |
|  | Total disagreement | Pretty much in disagreement | I don't know... | Pretty much in agreement | Total agreement |
| My child's team coach | 1 | 2 | 3 | 4 | 5 |
| A member of the local association | 1 | 2 | 3 | 4 | 5 |
| Other parents involved in half-ice hockey | 1 | 2 | 3 | 4 | 5 |
| A retired player from a high level of competition | 1 | 2 | 3 | 4 | 5 |
| Media | 1 | 2 | 3 | 4 | 5 |
| A member of the provincial association (Ontario Minor Hockey Association) | 1 | 2 | 3 | 4 | 5 |
| A member of the national federation (Hockey Canada) | 1 | 2 | 3 | 4 | 5 |
| A researcher specialized in this field | 1 | 2 | 3 | 4 | 5 |
|  | | | | | |

| 23. According to you, | | | | | |
| --- | --- | --- | --- | --- | --- |
|  | Total disagreement | Pretty much in disagreement | I don't know... | Pretty much in agreement | Total agreement |
| Half-ice hockey is more useful for most advanced players | 1 | 2 | 3 | 4 | 5 |
| If I had the choice, I would choose a half-ice games league for my child | 1 | 2 | 3 | 4 | 5 |
| I would prefer that my child progress in traditional full-ice hockey | 1 | 2 | 3 | 4 | 5 |
| I think the half-ice situation gives my child more chances to touch the puck | 1 | 2 | 3 | 4 | 5 |
| I think the half-ice game makes my child more likely to be involved in the game | 1 | 2 | 3 | 4 | 5 |
|  | | | | | |
